# Impact Of The Healthy, Hunger-Free Kids Act On Obesity Trends

**DOI:** 10.1377/hlthaff.2020.00133

**Published:** 2020-07

**Authors:** Erica L. Kenney, Jessica L. Barrett, Sara N. Bleich, Zachary J. Ward, Angie L. Cradock, Steven L. Gortmaker

**Affiliations:** Departments of Nutrition and Social and Behavioral Sciences at the Harvard T. H. Chan School of Public Health, in Boston, Massachusetts.; Department of Social and Behavioral Sciences, Harvard T. H. Chan School of Public Health.; Department of Health Policy and Management, Harvard T. H. Chan School of Public Health.; Center for Health Decision Science, Harvard T. H. Chan School of Public Health.; Department of Social and Behavioral Sciences, Harvard T. H. Chan School of Public Health.

## Abstract

The Healthy, Hunger-Free Kids Act of 2010 strengthened nutrition standards for meals and beverages provided through the National School Lunch, Breakfast, and Smart Snacks Programs, affecting fifty million children daily at 99,000 schools. The legislation’s impact on childhood obesity is unknown. We tested whether the legislation was associated with reductions in child obesity risk over time using an interrupted time series design for 2003–18 among 173,013 youth in the National Survey of Children’s Health. We found no significant association between the legislation and childhood obesity trends overall. For children in poverty, however, the risk of obesity declined substantially each year after the act’s implementation, such that obesity prevalence would have been 47 percent higher in 2018 if there had been no legislation. These results suggest that the Healthy, Hunger-Free Kids Act’s science-based nutritional standards should be maintained to support healthy growth, especially among children living in poverty.

Ensuring that children consume a healthful diet rich in fruit, vegetables, whole grains, and lean protein and low in added sugars and refined grains is a critical public health goal.^1^ Improving children’s nutrition can reduce their risk of obesity, which burdens 18.5 percent of two-to-nineteen-year-olds in the US, as well as the risk of future chronic disease.^2^ Policies shaping what foods and beverages are available in schools, which reach about fifty million US children and adolescents, provide an important opportunity to improve health, particularly for lower-income and minority children, who tend to have less access to healthy food^3^ and poorer dietary quality^4,5^ as well as a higher risk for obesity compared to other children.^6^

The passage of the Healthy, Hunger-Free Kids Act (HHFKA) in 2010 established a suite of policies to improve the nutritional quality of food and beverages served to US children through an array of federal food assistance programs. This included the National School Lunch Program, which affects thirty million students nationwide,^7^ and the School Breakfast Program, which affects fourteen million students nationwide.^8^ The National School Lunch Program began in 1946 and the School Breakfast Program, in 1996, to ensure that US children have access to nutritionally adequate meals during the school day. These programs are particularly essential for lower-income children, who participate at higher rates than other children and can receive free or reduced-price meals through them.^9^ However, the nutritional guidelines for meals and snacks served through these programs were originally developed long before childhood obesity and diet-related chronic disease had become a major concern, so they did not address limits on food and beverages that advances in nutrition science had found to be linked with excess weight gain.

Partly in an effort to help address the growing epidemic of childhood obesity, and after years of public health advocacy and research into optimal nutrition standards,^10^ the HHFKA changed the guidance for all meals and snacks provided through the National School Lunch Program and School Breakfast Program, aligning these programs—which had not been updated in over fifteen years^10^—with science-based recommendations from the National Academy of Medicine.^11^ Specifically, the meal patterns for breakfast and lunch changed to increase the amounts of fruit and vegetables served and limit starchy vegetables; create age-specific recommended serving sizes in recognition of differing calorie needs by age; serve only lowfat or fat-free milk; and serve more whole grains for grain products (only whole grains at lunch; half whole grains at breakfast).^12^ For the first time, the HHFKA also established standards for food and beverage products sold in schools outside of the breakfast and lunch programs (Smart Snacks), including á la carte offerings and snacks from vending machines or school stores. These Smart Snacks guidelines^13^ eliminated most sugary beverages and reduced the sugar and calorie content of food products for sale.

The US Department of Agriculture (USDA) began phasing in the HHFKA policy changes in the 2012–13 school year, and research suggests that these changes have been a public health success. Adherence to the new meal and snack standards has been high,^9,14^ and students consume more fruit, vegetables, and whole grains and fewer starchy vegetables than before the revision.^9,15^ At the same time, studies have found no increases in food waste^15–17^ or reductions in students’ participation in the National School Lunch Program.^18^

Despite these public health gains and implementation success, there has been substantial industry and political pushback to the HHFKA, with some organizations claiming that its nutrition standards for school meals and snacks must be weakened in order to reduce supposed food waste and compliance burdens.^10^ Within the past several years, whole-grain standards have been relaxed, although this rule change was recently vacated by a federal judge at the US District Court for the District of Maryland.^19^ Additional roll-backs, besides the whole-grain standards, have been proposed. A proposed rule published in January 2020 would allow schools to serve fewer nonstarchy fruits and vegetables and sell more pizza, hamburgers, and fries, among other changes.^20^

In light of these recent and proposed roll-backs, it is important to understand what impact the historic HHFKA changes to school nutrition standards may have had on childhood obesity, to shed light on what kinds of public health gains we might be giving up. Policies that could reduce childhood obesity are critical to identify: Apart from raising children’s risk for poor health in childhood, childhood obesity can also increase risk for adult obesity,^21^ as well as a range of debilitating and costly chronic diseases such as diabetes, cancer, and cardiovascular disease.^2^ While one study suggested slower excess weight gain for very young school lunch participants post-HHFKA, that particular study was far from conclusive, and it only addressed children up to third grade.^22^

Our goal with this study was to estimate whether the HHFKA changes reduced the public health burden of childhood obesity among a nationally representative sample of school-age children. Using large, nationally representative samples of ten-to-seventeen-year-olds collected between 2003 and 2018, this study estimates the extent to which populationwide trends in childhood obesity prevalence changed after the HHFKA’s changes to school meals and snacks. We used populationwide trends as a proxy for trends among school meal participants in this study. We hypothesized that time trends in obesity prevalence would begin decreasing after the first year of HHFKA implementation in 2012. Given higher school meal participation rates among children in poverty,^9^ we also hypothesized that children in poverty would see larger reductions in annual obesity prevalence trends compared to other children.

## Study Data And Methods

### STUDY DESIGN

We first estimated obesity prevalence trends among ten-to-seventeen-year-olds in all US states and the District of Columbia (hereafter, all states) from 2003 to 2018. We created repeated, cross-sectional estimates of obesity across six time points to evaluate whether trends in obesity prevalence changed after the Healthy, Hunger Free Kids Act’s school meal implementation began in fall 2012. We used an interrupted time series analysis approach^23^ and fit segmented regression models to test whether the time trend in having obesity significantly changed from before (time points including 2003, 2007, and 2011–12) to after (time points including 2016, 2017, and 2018) the time at which HHFKA implementation began. For more information on how time points were coded, see the online appendix.^24^

### SAMPLE

We leveraged data from the National Survey of Children’s Health, a large, periodic, nationally representative survey of noninstitutionalized children ages 0–17 conducted in all states. The survey has been conducted annually since 2016; prior to that, it was conducted in 2003, 2007, and 2011–12. For all years a multistage sampling design was used, with the sample stratified by state, households selected randomly within states, and one child selected randomly per household.^25^ A parent or guardian of the sampled child with knowledge of the child’s health and health care was then asked to complete a survey about that child. For this analysis we used survey responses from 2003, 2007, 2011–12, 2016, 2017, and 2018.We included participants ages 10–17 with nonmissing data on weight status and sociodemographic variables described below. Weight status is not reported for children younger than age ten in the National Survey of Children’s Health public-use data files from 2007 to 2018 because of reported validity concerns.^26^ For more information on the sampling procedures for that survey, see the appendix.^24^

### MEASURES

#### OUTCOME VARIABLE:

The primary outcome for this study was obesity, defined as having a body mass index (BMI) above the ninety-fifth percentile for a child’s age and biological sex according to the 2000 Growth Charts of the Centers for Disease Control and Prevention.^27^ The parent or guardian respondent reported the child’s weight and height. Survey staff then calculated BMI, compared to the growth chart percentiles, and classified each child as underweight (below the fifth percentile), healthy weight (from the fifth to below the eighty-fifth percentiles), overweight (from the eighty-fifth to below the ninety-fifth percentiles), and obesity (ninety-fifth percentile and above).

#### INDIVIDUAL-LEVEL COVARIATES:

Demographic covariates at the child level included age, biological sex, race/ethnicity (non-Hispanic white, non-Hispanic black, Hispanic/Latino, and non-Hispanic other), and the poverty status of the household (at or below 100 percent of the federal poverty level), which were all reported by the parent or guardian or derived from reports of family income.

#### STATE-LEVEL COVARIATES:

To control for the possibility that any observed trends might be influenced by preexisting state-level nutrition policies, rather than the introduction of the federal-level HHFKA policies, we leveraged data on state-level school nutrition policies from the Classification of Laws Associated with School Students (CLASS) database.^28^ This database includes variables representing the strength of nutrition policies in schools for 2003–15 across several domains. We classified states according to whether or not they had strong nutrition standards for school meals before the implementation of the HHFKA policies and whether or not they had standards for food sold outside of school meals, consistent with the Smart Snacks guidelines specified by the HHFKA. For more details on the CLASS scoring, see the appendix.^24^

### STATISTICAL ANALYSIS

To conduct the interrupted time series analysis, we fit segmented multivariable logistic regression models adjusting for the complex sampling design. The models predicted the odds of a study participant having obesity as a function of time in years (centered at fall 2012, the HHFKA’s first year of implementation for school meals) and an additional term for time after the introduction of the HHFKA policies (post intervention—that is, 2016, 2017, and 2018 only), which tested whether there was a change in the time trend after the introduction of those policies. The resulting odds ratio (OR) estimates for the time variable year represent the change in the odds of a ten-to-seventeen-year-old having obesity from one year to the next from 2003 to 2018. The resulting OR for time after the introduction of the HHFKA policies represents the average annual change in the trend for odds of obesity for each year after 2012 (a change in slope). In other words, the “time after HHFKA” coefficient tests whether the yearly trend in obesity risk changes after 2012.

To account for how changes in the sociodemographic makeup of the US adolescent population could have affected time trends in obesity risk, we adjusted for survey participants’ race/ethnicity, household poverty status, age, and biological sex. We also controlled for preexisting state policies on school meals (in 2010) and food products sold outside the school meal program (in 2013). To account for state-level variation in obesity prevalence trends, we included fixed effects for every state, using state indicator variables.

To test for whether the trends differed according to a child’s poverty status, as poverty is associated with a higher likelihood of eating school meals^9^ and thus may be associated with a larger likelihood of benefitting from the HHFKA, we fit models that included the covariates above as well as interaction terms for poverty status and the pre-HHFKA trend plus poverty status and the post-HHFKA trend.

We estimated uncertainty for model estimates using 100 sets of replicate weights estimated by bootstrapping the data set while accounting for the complex survey design in each survey round.^29^ We also calculated the predicted probability of having obesity for each year and by poverty status.

All models were estimated using PROC SURVEYLOGISTIC in SAS, version 9.4.

### LIMITATIONS

Although this study benefited from leveraging several years of nationally representative population-level data to understand time trends in childhood obesity, there are several limitations that preclude us from definitively attributing any changes in obesity to the HHFKA. First, the data points for estimating the post-HHFKA time trend are relatively few and close together (2016, 2017, and 2018), given how recently the policies were implemented. This made our estimation of the change in trend less reliable; also, it did not allow us to examine changes in obesity prevalence during different phases of implementation, including the phasing in and rolling back of standards.

Second, the National Survey of Children’s Health did not include information on study participants’ own consumption of school meals and snacks, and thus we were not able to identify who was and was not consuming school meals; this may have led us to an underestimate of the potential impact of the HHFKA.

Third, because the HHFKA is a federal law whose policies cover food and beverages served in all schools participating in the National School Lunch Program, we did not have a separate comparison group that was not exposed to the policies to test whether the observed changes in trends were due to other factors, although there were no other events or policy shifts occurring during the relevant time period tested that would serve as alternative explanations for any observed changes.

Fourth, the measures of height and weight from the National Survey of Children’s Health that are used to calculate each participant’s weight status are collected via parental report, which is subject to bias,^30^ but this should not change over time and thus should not affect time trend estimates.

Last, the study is limited in only being able to assess changes among ten-to-seventeen-year-olds, given the absence of weight class data for younger children in the National Survey of Children’s Health.

## Study Results

Across the six survey periods of the National Survey of Children’s Health (2003, 2007, 2011–12, 2016, 2017, and 2018), there were 193,370 participants ages 10–17. Of these, we excluded 20,357 participants because of missing data on BMI, poverty, or race/ethnicity (10.5 percent of the original sample), for a final sample of 173,013. Earlier survey waves had larger sample sizes as a result of the different survey design (every four years rather than every year, as has been in place since 2016) (exhibit 1). The mean age of participants across all years was 13.5, with little variation across years. Similarly, the sample was 51 percent male for all survey periods. Race/ethnicity varied across survey periods, with the share of survey participants identifying as non-Hispanic white dropping steadily over time, from 66.1 percent in 2003 to 50.2 percent in 2018, and those identifying as Hispanic (any race) increasing from 11.9 percent to 26.6 percent over the same time period. The share of the population identified as living in poverty also varied across survey years, increasing from 14.8 percent in 2003 to 20.0 percent in 2016, then decreasing to 16.9 percent by 2018. The prevalence of obesity in the population fluctuated in the range of 15–16 percent across survey years, with the lowest estimates in 2003 and 2018. Prior to the implementation of the HHFKA, just four states (7.8 percent) had preexisting regulations specifying nutrition criteria for National School Lunch Program meals similar to those in the HHFKA, while eleven (21.6 percent) had regulations specifying strong nutrition criteria for food products sold outside of school meal programs (data not shown).

Adjusting for children’s age, sex, race/ethnicity, and poverty status, as well as state fixed effects, and accounting for the complex sampling design, we found that before the HHFKA’s school meal and snack standards took effect, there was no meaningful time trend in the likelihood of having obesity (OR for an change in obesity for each year: 1.01; *p* > 0:05) and no significant evidence for a change in the risk of having obesity after the implementation of the new HHFKA standards (OR: 0.98; *p* > 0:05) (model 1 in exhibit 2). Adding controls for preexisting state-level nutrition policies for school meals and food products sold outside of school meal programs had no impact on these estimates and thus were not included in the final model. Similarly, when testing for whether pre- and post-HHFKA time trends differed by state policy status, we found no significant results (see the appendix).^24^

For children in poverty, however, we found that prior to the HHFKA’s changes to school meals and snacks, the odds of having obesity had been increasing year after year (OR: 1.04 per year; *p* = 0:003), while after the HHFKA’s implementation, the yearly trend in the odds of having obesity began decreasing (OR: 0.91; *p* = 0:004) (model 2 in exhibit 2). In other words, after the HHFKA was implemented for school meals, children in poverty had a 9 percent lower odds of having obesity each year, when the the other variables were controlled for. In 2018 the predicted probability of obesity for children in poverty was approximately 0.21 with the HHFKA but would have been expected to be 0.31 had the time trends prior to the HHFKA continued—in other words, the risk of obesity would have been 47 percent higher in 2018 without the legislation (exhibit 3). Exact *p* values and 95% confidence intervals for all model estimates are in the appendix.^24^

## Discussion

This study, using nationally representative data of 173,013 children from all states over a fifteen-year period suggests that the passage of the Healthy, Hunger-Free Kids Act and implementation of its changes to school meals and snacks— currently affecting children in more than 99,000 schools across the US—was associated with significantly decreased risk of obesity for the estimated 5.9 million US children ages 10–17 in poverty.^31^ These are the children who, because of their higher levels of participation in school meals, stand to benefit most from the HHFKA.^9^ After the HHFKA’s implementation for school meals and snacks, youth in poverty—who are particularly vulnerable to obesity^6^—saw their odds of having obesity reduced by 9 percent annually; by 2018 their risk of obesity would have been 47 percent higher if there had been no legislation. Roughly, we estimate that in 2018 this meant over 500,000 fewer cases of obesity among children in poverty, reducing the risk of future chronic diseases for these children as well as avoiding substantial health care costs.^32^ These results were robust to adjustment for changes in the sociodemographic makeup of the US youth population. There was no change in risk for children not living in households in poverty.

We are unable to definitively state a causal relationship between the HHFKA and the reversal in childhood obesity prevalence trends among children in poverty, because of the infeasibility of using a randomized design for such a policy evaluation and because the data set did not have a variable to explicitly indicate participation in school meals. However, our finding of a decrease in obesity risk among children in poverty is supported by findings of changes in dietary intake elsewhere. Studies evaluating the impact of the HHFKA’s school meal changes on dietary intake have found that students eating school meals consume fewer total calories and more fruit, vegetables, and whole grains than prior to the HHFKA, as well as fewer starchy vegetables.^15^ These dietary changes have been clearly linked with weight loss or reductions in excess weight gain.^33,34^ Additionally, we are simply unaware of another policy at the same nationwide scale impacting children in poverty that could be a likely explanation for a shift in obesity risk among youth in this age group. Changes to the nutrition standards of the Special Supplemental Program for Women, Infants, and Children (WIC) in 2009 were found to reduce risk of obesity for low-income children ages 2–4;^35,36^ however, the children affected by that policy change would not enter as ten-year-olds in the National Survey of Children’s Health samples until 2015.

Results from this analysis suggest that the HHFKA school meal and snack standards may be helping reduce the risk of obesity among children in poverty and should be maintained, if not further strengthened. Indeed, given the recent attempts to relax the HHFKA standards, particularly the attempted weakening of a requirement for serving whole grains, as well as recently proposed weakening of fruit and vegetable requirements,^20^ it is possible that the gains seen here could diminish in the future. These rollback efforts should be reconsidered, particularly because they were largely grounded in concerns about increased food waste and infeasible implementation, concerns that scientific research suggests are unfounded, as there have been no changes in food waste,^15,16^ and implementation of the new standards has been high, with over 80 percent of schools meeting the standards.^9^ These results also suggest that since the beneficial change to obesity risk did not extend to children not in poverty, policy makers could consider strategies for increasing participation in school meals among students who are not currently eligible for free or reduced-price lunch.

## Conclusion

The implementation of stronger nutrition standards for school meals and snacks through the Healthy, Hunger-Free Kids Act was associated with a significant reduction in the risk of obesity for youth in poverty. The original 2010 HHFKA standards should be restored, and efforts to increase participation should be strengthened, to build on the law’s progress in reducing childhood obesity in the United States.

## Supplementary Material

Appendix

Erratum

## Figures and Tables

**EXHIBIT 3:**
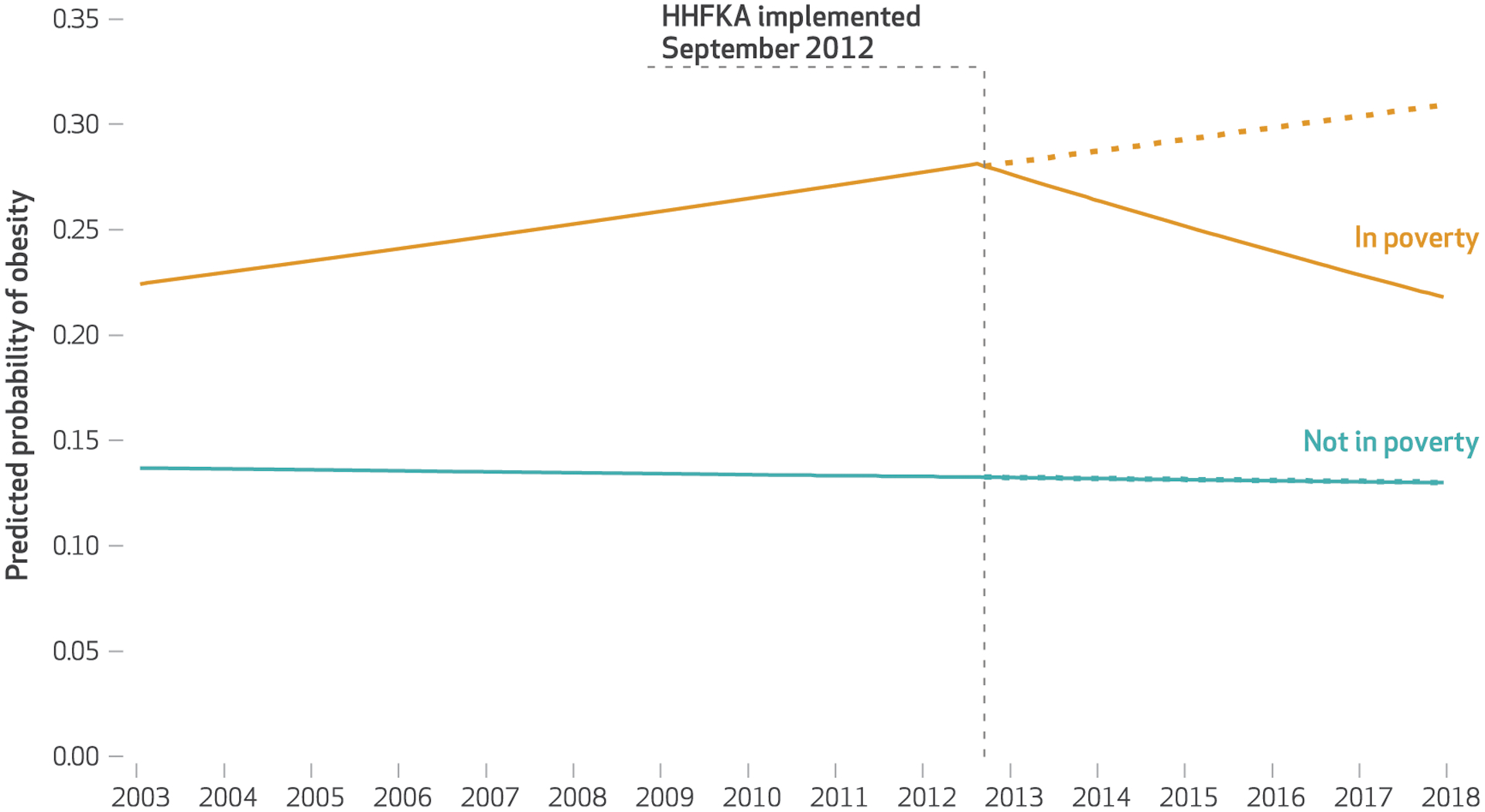
Predicted probability of obesity among youth ages 10–17 before and after implementation of Healthy, Hunger Free Kids Act (HHFKA) changes to the National School Lunch Program, by poverty status, 2003–18 **SOURCE** Authors’ analysis of data from the National Survey of Children’s Health, 2003–18. **NOTES** Sample includes youth ages 10–17 with reported body mass index, poverty status, race, and ethnicity. Survey responses from 2003, 2007, 2011–12, 2016, 2017, and 2018 were used for this analysis. Predicted probability represents the average weighted value from the sample and is derived from weighted logistic regression models that adjust for participant age, sex, race/ethnicity, and state of residence. Dotted lines show pre-HHFKA trends projected post-HHFKA, for youth in poverty and not in poverty. “Not in poverty” indicates family income above 100 percent of the federal poverty level. “In poverty” indicates family income at or below the federal poverty level.

**EXHIBIT 1:** Descriptive characteristics of the National Survey of Children’s Health sample for each survey wave, selected years 2003–18

	Pre-HHFKA						Post-HHFKA					
	2003 (*n* = 42,417)		2007 (*n* = 40,364)		2011–12 (*n* = 39,561)		2016 (*n* = 24,405)		2017 (*n* = 10,839)		2018 (*n* = 15,427)	
Characteristics	Number	SE/%^a^	Number	SE/%^a^	Number	SE/%^a^	Number	SE/%^a^	Number	SE/%^a^	Number	SE/%^a^
Mean age, years (SE)	13.5	(0.02)	13.6	(0.03)	13.5	(0.03)	13.5	(0.03)	13.6	(0.05)	13.5	(0.04)
Male	21,889	50.8	21,017	50.7	20,661	51.5	12,371	51.1	5,541	51.1	8,110	51.2
Race/ethnicity												
Non-Hispanic white	31,456	66.1	28,985	59.8	27,569	56.9	17,555	53.7	7,585	51.0	10,901	50.2
Non-Hispanic black	4,096	15.2	4,062	15.2	3,749	14.6	1,364	12.8	724	14.6	998	13.5
Hispanic	3,861	11.9	4,019	17.1	4,341	19.2	2,556	23.9	1,187	24.6	1,756	26.6
Non-Hispanic other	3,004	6.8	3,298	8.0	3,902	9.3	2,930	9.7	1,343	9.8	1,772	9.7
In poverty	4,120	14.8	3,797	14.5	4,818	17.8	2,014	20.0	1,050	16.5	1,386	16.9
Weight status												
Underweight	1,989	4.8	1,983	5.1	2,246	5.9	1,491	6.3	697	6.2	1,004	7.3
Healthy weight	28,125	64.6	26,639	63.1	25,691	62.4	16,339	62.6	7,174	63.1	10,136	61.9
Overweight	6,460	15.8	6,196	15.4	5,901	15.8	3,474	15.0	1,533	15.3	2,258	15.7
Obesity	5,843	14.8	5,546	16.4	5,723	15.9	3,101	16.1	1,435	15.4	2,029	15.1

**SOURCE** Authors’ analysis of data from the National Survey of Children’s Health, selected years 2003–18. **NOTES** Sample includes youth ages 10–17 with reported body mass index, poverty status, and race/ethnicity. HHFKA is Healthy, Hunger-Free Kids Act.

a Standard error or weighted percentage. Values are percentages except where indicated (age).

**EXHIBIT 2:** Change per year in the odds of having obesity before and after implementation of Healthy, Hunger-Free Kids Act (HHFKA) changes to the National School Lunch Program

Variables	Odds ratios	
	Model 1: overall effects	Model 2: effects by poverty status
Time (years)	1.01	1.00
Time (years) after HHFKA	0.98*	1.00
Time (years) for children in poverty	—^a^	1.04***
Time (years) after HHFKA for children in poverty	—^a^	0.91***
Demographic characteristics controlled for in		
estimating obesity prevalence time trends		
In poverty (versus not in poverty)	1.62****	2.05****
Age (years, continuous)	0.93****	0.93****
Male (versus female)	1.44****	1.44****
Race/ethnicity		
Non-Hispanic black	1.88****	1.87****
Hispanic/Latino	1.79****	1.79****
Non-Hispanic other	1.09	1.09
Non-Hispanic white	Ref	Ref

**SOURCE** Authors’ analysis of data from the National Survey of Children’s Health, 2003–18. **NOTES** Sample includes youth ages 10–17 with reported body mass index, poverty status, race, and ethnicity. Survey responses from 2003, 2007, 2011–12, 2016, 2017, and 2018 were used for this analysis. Weighted logistic regression models were used and were also adjusted for state. Model 1 examined changes overall, and model 2 examined changes by child poverty status. “In poverty” was defined as living in a family with income at or below 100 percent of the federal poverty level. Confidence intervals and exact p values are in the online appendix (see note 25 in text).

a Variables were not considered in model 1.

* *p* < 0.10

*** *p* < 0.01

**** *p* < 0.001
