# Supplementary material for: Impact Of The Healthy, Hunger-Free Kids Act On Obesity Trends
Source: Health Aff (Millwood). Author manuscript; Available in PMC 2021 Mar 16. (PMC7961790; doi:10.1377/hlthaff.2020.00133)
Supplement: Appendix [file NIHMS1672883-supplement-Appendix.pdf]

Appendix

Table of Contents:

- **Appendix A1. Sampling approaches and time coding for the National Survey of Children's Health**
  - **APPENDIX EXHIBIT A1.1**. National Survey of Children's Health (NSCH) data collection periods and time coding used in the analysis of obesity prevalence before and after implementation of the Healthy, Hunger-Free Kids Act (HHFKA) changes to the National School Lunch Program.
- **Appendix A2. State-level school nutrition policies**
  - **APPENDIX EXHIBIT A2.1**. Change per year in the odds of having obesity before and after implementation of the Healthy, Hunger-Free Kids Act (HHFKA) changes to the National School Lunch Program, adjusting for pre-HHFKA state-level school nutrition standard policies.
- **Appendix A3. Estimates of uncertainty for time trends in the likelihood of having obesity before and after implementation of the Healthy, Hunger-Free Kids Act (HHFKA) changes to the National School Lunch Program.**
  - **APPENDIX EXHIBIT A3.1.** Change per year in the odds of having obesity before and after implementation of the Healthy, Hunger-Free Kids Act (HHFKA) changes to the National School Lunch Program, including estimates of uncertainty.

Appendix A1. Sampling approaches and time coding for the National Survey of Children's Health

Sampling Notes. In 2003 and 2007 landline phone surveys were used.(1) For 2011/2012, the sample was drawn from landline or cellphone records.(2) From 2016 onward, a slightly different approach was used. Addresses were randomly sampled, with oversampling of addresses determined to be more likely to have 0-17 year old children residing in them and one child was sampled at random per household.(3) NSCH sample sizes also decreased in recent years relative to earlier waves; however, apart from changes in racial/ethnic makeup that reflected changes in the population at large,(4) there were no significant differences in the demographics of the samples when comparing earlier to later waves, suggesting the smaller recent samples were still likely representative.

In our analysis, we coded the timing of each survey wave according to the midpoint of the survey period (see **APPENDIX EXHIBIT A1.1**).

APPENDIX EXHIBIT A1.1. National Survey of Children's Health (NSCH) data collection periods and time coding used in the analysis of obesity prevalence before and after implementation of the Healthy, Hunger-Free Kids Act (HHFKA) changes to the National School Lunch Program.

Survey Wave	Data Collection Dates	Time (Years) Coding Used in Analysis
2003	January 2003 through July 2004(1)	2003.79 (September/October 2003)
2007	April 2007 through July 2008(5)	2007.92 (November 2007)
2011/2012	February 2011 through June 2012(2)	2011.79 (September/October 2011)
2016	June 2016 through January 2017(6)	2016.75 (September 2016)
2017	August 2017 through February 2018(7)	2017.88 (October/November 2017)
2018	June 2018 through January 2019(8)	2018.75 (September 2018)

SOURCE Authors' analysis of data from the National Survey of Children's Health, 2003-2018. NOTES Time in years used in analysis was coded according to the midpoint of data collection periods for each NSCH survey wave.

Appendix A2. State-level school nutrition policies

Data on state-level school nutrition policies from the Classification of Laws Associated with School Students (CLASS) database(9) were used to classify states according to the strength of nutrition standards for school meals and foods sold outside of school meals prior to HHFKA. States were classified as having strong pre-HHFKA nutrition standards for school meals if, in 2010, their CLASS scores for policies for school meals indicated they were already consistent with the HHFKA guidelines (a score of 3 or above for the “Reimbursable School Lunch Requirements” variable(9)). States were also classified as having strong standards for foods sold outside of school meals if, in 2013, their CLASS scores were consistent with the Smart Snacks guidelines specified by HHFKA. We used average CLASS scores across elementary, middle, and high school grade levels in 2013 averaged across a la carte, school stores, vending, and other venues for purchasing foods and beverages outside the school meal program. We then coded a state as having had a pre-existing policy similar to Smart Snacks if their average score was greater than 4(9).

We adjusted for these pre-existing policies in weighted logistic regression models examining change per year in the odds of having obesity before and after implementation of the HHFKA changes to the National School Lunch Program. We examined the differences in average obesity prevalence in states with or without pre-existing policies, as well as the trends in obesity prevalence before and after implementation of the HHFKA school nutrition standards in states with and without pre-existing policies.

The state-level nutrition policies for school meals and foods sold outside of school meals had no significant independent impact on obesity trends (**APPENDIX EXHIBIT A2.1**).

APPENDIX EXHIBIT A2.1. Change per year in the odds of having obesity before and after implementation of the Healthy, Hunger-Free Kids Act (HHFKA) changes to the National School Lunch Program, adjusting for pre-HHFKA state-level school nutrition standard policies.

	Odds Ratio	*95% Confidence Interval for Odds Ratio*		*p-value*
Time (years)	1.01	1.00	1.02	0.30
Time (years) after HHFKA	0.98	0.95	1.00	0.07
Time (years) for states with pre-existing school meals standards	1.03	1.00	1.06	0.07
Time (years) after HHFKA for states with pre-existing school meals standards	0.98	0.90	1.05	0.53
Time (years) for states with pre-existing Smart Snacks standards	0.99	0.97	1.02	0.55
Time (years) after HHFKA for states with pre-existing Smart Snacks standards	1.01	0.95	1.07	0.71
Demographic Characteristics Controlled for in Estimating Obesity Prevalence Time Trends				
Poverty (vs. Not in Poverty)	1.62	1.46	1.80	<.0001
Age (years)	0.93	0.91	0.94	<.0001
Male (vs. Female)	1.44	1.33	1.57	<.0001
Race/Ethnicity				
Non-Hispanic Black	1.88	1.69	2.08	<.0001
Hispanic/Latino	1.79	1.60	2.01	<.0001
Non-Hispanic Other Race	1.09	0.97	1.24	0.16
Non-Hispanic White	Reference			
Pre-HHFKA State-Level School Nutrition Standard Policies				
State had pre-existing policy for NSLP meals aligned with HHFKA standards	0.94	0.70	1.27	0.69
State had pre-existing policy aligned with Smart Snacks standards	1.45	1.15	1.84	0.002

SOURCE Authors' analysis of data from the National Survey of Children's Health, 2003-2018. NOTES Sample includes youth ages 10-17 years with reported body mass index, poverty status, race, and ethnicity. Survey responses from 2003, 2007, 2011/2012, 2016, 2017, and 2018 were utilized for this analysis. Weighted logistic regression models were used and were also adjusted for state. Model 1 examined changes overall, and Model 2 examined changes by child poverty status. Poverty was defined as living in a family with income less than 100% of the federal poverty level. Uncertainty around model estimates is reported as 95% confidence intervals and exact p-values, which were obtained using 100 sets of replicate weights estimated by bootstrapping the dataset while accounting for the complex survey design in each survey round.

Appendix A3. Estimates of uncertainty for time trends in the likelihood of having obesity before and after implementation of the Healthy, Hunger-Free Kids Act (HHFKA) changes to the National School Lunch Program.

We estimated uncertainty around model estimates (95% confidence intervals) using 100 sets of replicate weights estimated by bootstrapping the dataset while accounting for the complex survey design in each survey round.(10) All models were estimated using PROC SURVEYLOGISTIC in SAS v. 9.4 (Cary, NC: SAS Institute). Confidence intervals and exact p-values for weighted logistic regression model results presented in the manuscript are displayed below (**APPENDIX EXHIBIT 3.1**).

APPENDIX EXHIBIT A3.1. Change per year in the odds of having obesity before and after implementation of the Healthy, Hunger-Free Kids Act (HHFKA) changes to the National School Lunch Program, including estimates of uncertainty.

	Model 1: Overall effect				*Model 2: Effects by poverty status*			
	Odds Ratio	*95% Confidence Interval for Odds Ratio*		*p-value*	*Odds Ratio*	*95% Confidence Interval for Odds Ratio*		*p-value*
Time (years)	1.01	1.00	1.01	0.32	1.00	0.99	1.01	0.48
Time (years) after HHFKA	0.98	0.95	1.00	0.055	1.00	0.97	1.03	0.97
Time (years) for children in poverty					1.04	1.01	1.06	0.003
Time (years) after HHFKA for children in poverty					0.91	0.85	0.97	0.004
Demographic Characteristics Controlled for in Estimating Obesity Prevalence Time Trends								
Poverty (vs. Not in Poverty)	1.62	1.46	1.80	<.0001	2.05	1.77	2.38	<.0001
Age (years)	0.93	0.91	0.94	<.0001	0.93	0.91	0.94	<.0001
Male (vs. Female)	1.44	1.33	1.57	<.0001	1.44	1.33	1.57	<.0001
Race/Ethnicity								
Non-Hispanic Black	1.88	1.69	2.08	<.0001	1.87	1.69	2.08	<.0001
Hispanic/Latino	1.79	1.60	2.01	<.0001	1.79	1.59	2.01	<.0001
Non-Hispanic Other Race	1.09	0.96	1.24	0.16	1.09	0.96	1.24	0.17
Non-Hispanic White	Reference				Reference			

SOURCE Authors' analysis of data from the National Survey of Children's Health, 2003-2018. NOTES Sample includes youth ages 10-17 years with reported body mass index, poverty status, race, and ethnicity. Survey responses from 2003, 2007, 2011/2012, 2016, 2017, and 2018 were utilized for this analysis. Weighted logistic regression models were used and were also adjusted for state. Model 1 examined changes overall, and Model 2 examined changes by child poverty status. Poverty was defined as living in a family with income less than 100% of the federal poverty level. Uncertainty around model estimates is reported as 95% confidence intervals and exact p-values, which were obtained using 100 sets of replicate weights estimated by bootstrapping the dataset while accounting for the complex survey design in each survey round.

Appendix References

1. Blumberg SJ, Olson L, Frankel MR, Osborn L, Srinath KP, Giambo P. Design and Operation of the National Survey of Children’s Health, 2003. Vital Health Stat 1. 2005 Jun;(43):1–131.
2. Bramlett MD, Blumberg SJ, Zablotsky B, George JM, Ormson AE, Frasier AM, et al. Design and Operation of the National Survey of Children’s Health, 2011-2012. Vital Health Stat 1. 2017 Jul;(59):1–256.
3. Data Resource Center for Child and Adolescent Health. NSCH Survey Methodology [Internet]. 2019 [cited Jan 9 2020]. Available from: https://www.childhealthdata.org/learn-about-the-nsch/methods
4. Racial and Ethnic Composition of the Child Population [Internet]. 2018 [cited Jan 9 2020]. Available from: https://www.childtrends.org/indicators/racial-and-ethnic-composition-of-the-child-population
5. Blumberg SJ, Foster EB, Frasier AM, Satorius J, Skalland BJ, Nysse-Carris KL, et al. Design and operation of the National Survey of Children’s Health, 2007. Vital Health Stat 1. 2012 Jun;(55):1–149.
6. U.S. Census Bureau. 2016 National Survey of Children’s Health: Methodology Report. [Internet]. 2018 [cited 2020 Apr 9]. Available from: https://www.census.gov/content/dam/Census/programs-surveys/nsch/tech-documentation/methodology/2016-NSCH-Methodology-Report.pdf
7. U.S. Census Bureau. 2017 National Survey of Children’s Health: Methodology Report [Internet]. 2018 [cited 2020 Apr 9]. Available from: https://www.census.gov/content/dam/Census/programs-surveys/nsch/tech-documentation/methodology/2017-NSCH-Methodology-Report.pdf
8. U.S. Census Bureau. 2018 National Survey of Children’s Health: Methodology Report [Internet]. 2019 [cited 2020 Apr 9]. Available from: https://www2.census.gov/programs-surveys/nsch/technical-documentation/methodology/2018-NSCH-Methodology-Report.pdf
9. National Cancer Institute. Classification of Laws Associated with School Students (CLASS) [Internet]. 2016. Available from: https://class.cancer.gov/index.aspx
10. Asparouhov T, Muthen B. Resampling Methods in Mplus for Complex Survey Data [Internet]. 2010 [cited Jan 9 2020]. Available from: https://www.statmodel.com/download/Resampling_Methods5.pdf
