# Supplementary material for: Impact Of The Healthy, Hunger-Free Kids Act On Obesity Trends
Source: Health Aff (Millwood). Author manuscript; Available in PMC 2021 Mar 16. (PMC7961790; doi:10.1377/hlthaff.2020.00133)
Supplement: Erratum [file NIHMS1672883-supplement-Erratum.pdf]

Erratum

2020-00133 KENNEY ET AL., 39:7, PP. 1122, 1127, 1128 An author error in phrasing the interpretation of some of the findings has resulted in four changes to the text, none of which affect the article's findings or conclusions. In the abstract, a passage of text, "translating to a 47 percent reduction in obesity prevalence in 2018 from what would have been expected without the legislation," now reads, "such that obesity prevalence would have been 47 percent higher in 2018 if there had been no legislation." In the paragraph above the subhead "Discussion," a passage of text, "in other words, a 47 percent reduction in the predicted probability of obesity," now reads, "in other words, the risk of obesity would have been 47 percent higher in 2018 without the legislation." In the first paragraph below "Discussion," a passage of text, "translating to a 47 percent reduction in obesity prevalence in 2018 from what would have been expected without the HHFKA," now reads, "by 2018 their risk of obesity would have been 47 percent higher if there had been no legislation." In the final paragraph, a passage of text, "a 47 percent reduction in obesity prevalence in 2018 from what would have been expected without that legislation," has been deleted. The article has been corrected online

Downloaded from HealthAffairs.org on February 11, 2021.
Copyright Project HOPE—The People-to-People Health Foundation, Inc.
For personal use only. All rights reserved. Reuse permissions at HealthAffairs.org.
